# Effect of 26 Weeks of Liraglutide Treatment on Coronary Artery Inflammation in Type 2 Diabetes Quantified by [^64^Cu]Cu-DOTATATE PET/CT: Results from the LIRAFLAME Trial

**DOI:** 10.3389/fendo.2021.790405

**Published:** 2021-11-30

**Authors:** Jacob K. Jensen, Emilie H. Zobel, Bernt J. von Scholten, Viktor Rotbain Curovic, Tine W. Hansen, Peter Rossing, Andreas Kjaer, Rasmus S. Ripa

**Affiliations:** ^1^ Department of Clinical Physiology, Nuclear Medicine and PET & Cluster for Molecular Imaging, Copenhagen University Hospital – Rigshospitalet & Department of Biomedical Sciences, University of Copenhagen, Copenhagen, Denmark; ^2^ Steno Diabetes Center Copenhagen, Gentofte, Denmark; ^3^ Novo Nordisk A/S, Søborg, Denmark

**Keywords:** inflammation, coronary arteries, PET, type 2 diabetes, molecular imaging, atherosclerosis

## Abstract

**Background:**

Quantification of coronary artery inflammation and atherosclerosis remains a challenge in high-risk individuals. In this study we sought to investigate if the glucagon like peptide-1 receptor agonist liraglutide has a direct anti-inflammatory effect in the coronary arteries using positron emission tomography (PET) with a radioactive tracer targeting activated macrophages in the vessel-wall.

**Methods:**

Thirty randomly selected participants with type 2 diabetes from the placebo-controlled trial LIRAFLAME were enrolled in this sub-study. Participants were, prior to enrollment in this sub-study, randomized to either treatment with daily liraglutide (n=15) or placebo (n=15). Both groups underwent a combined [^64^Cu]Cu-DOTATATE positron emission tomography and computed tomography scan of the heart at baseline and after 26 weeks of treatment. Coronary artery uptake of [^64^Cu]Cu-DOTATATE were measured as maximum standardized uptake values (SUV_max_); and means of the maximum values (mSUV_max_), both values were calculated at the level of each participant and each individual coronary-segment.

**Results:**

SUV_max_ and mSUV_max_ values decreased significantly in the liraglutide group both at the participant level (SUV_max_: p=0.013; mSUV_max_: p=0.004) and at the coronary-segment level (SUV_max_: p=0.001; mSUV_max_: p<0.0001). No change was observed in the placebo group neither at the participant level (SUV_max_: p=0.69; mSUV_max_: p=0.67) or at the coronary-segment level (SUV_max_: p=0.49; mSUV_max_: p=0.30). When comparing the mean change in uptake values between the two groups at both the participant level (SUV_max_: p=0.076; mSUV_max_: p=0.077) and the coronary segment level (SUV_max_: p=0.13; mSUV_max_: p=0.11) a borderline significant difference was observed. Baseline SUV_max_ [^64^Cu]Cu-DOTATATE uptake values showed a weak positive correlation with the inflammatory biomarker high-sensitivity c-reactive protein (τ =0.26, p=0.045).

**Conclusion:**

Liraglutide treatment for 26-weeks caused a significant reduction in [^64^Cu]Cu-DOTATATE uptake in the coronary arteries whereas this was not seen in the placebo treated group. In addition, [^64^Cu]Cu-DOTATATE PET/CT as a marker of coronary inflammation correlated with the systemic inflammation marker hs-CRP.

## Introduction

Inflammatory mediated coronary atherosclerosis is the primary cause of plaque progression and myocardial infarction, and a major contributor to cardiovascular morbidity and mortality in the western world (1).

Non-invasive imaging of coronary atherosclerosis using computed tomography (CT) coronary artery calcium score (CACS) has for a number of years been a staple for estimating individual risk of cardiovascular events (2). However, CACS has certain limitations and is not able to detect ongoing active inflammation in the vessel wall. Positron emission tomography (PET) for detection of active atherosclerotic inflammation is fast becoming an established technique that allows for *in vivo* quantification of the inflammatory plaque burden. The most commonly used PET-tracer [^18^F]Fluoro-deoxy-glucose ([^18^F]FDG) has shown promise in the evaluation of treatment effects of drugs aimed at reducing vascular inflammation e.g. statins in clinical trials (3, 4). Even though [^18^F]FDG PET imaging of atherosclerosis has been extensively evaluated in large- and mid-sized arteries it presents a unique problem when imaging the coronary arteries; myocardial spillover of the tracer, leading to difficulties in discriminating uptake in the coronary arteries, from that of the myocardium. Therefore, alternative molecular imaging probes are continuously being evaluated for their use in coronary atherosclerosis. [1,4,7,10-tetraazacyclododecane-N,N’,N’’,N’’’-tetraacetic acid]-D-Phe1, Tyr3-octreotate (DOTATATE) a ligand for the somatostatin receptor subtype-2 (SSTR_2_), is routinely used in the diagnosis and staging of patients with neuroendocrine tumors. As SSTR_2_ is also expressed on activated M1 macrophages, DOTATATE may therefore offer a superior cell-specificity compared to [^18^F]FDG (5).

People with type 2 diabetes are known to have a higher risk of cardiovascular disease than the general population (6). In recent years multiple novel therapies for the treatment and management of type 2 diabetes have emerged, among these are the glucagon like peptide-1 receptor agonists (GLP-1RA). Liraglutide, a once-daily GLP-1RA has, along with other drugs of the same class, shown to be efficacious in reducing the cardiovascular risk of high-risk groups in clinical outcome trials (7–10), and multiple animal studies have shown a delay and augmentation in plaque formation (11–13). Previously, we conducted a randomized placebo controlled clinical trial (LIRAFLAME), which aimed to investigate if reduction in vessel wall inflammation measured as uptake of [^18^F]FDG in the carotid arteries and aorta could explain the reduction of cardiovascular events in patients administered liraglutide compared to placebo (14). Here we found no effect of liraglutide on the primary outcome in 102 participants, although exploratory analysis in participants with known cardiovascular disease indicated a possible effect.

A sub-study using 30 participants from the LIRAFLAME study-population measuring vascular inflammation in the carotid arteries using [^64^Cu]Cu-DOTATATE showed a non-significant reduction of uptake in the liraglutide treated group compared to placebo (15). However, the coronary arteries might be a more appropriate target organ than the carotid arteries for assessing vascular inflammation, due to the fact that coronary disease accounts for the majority of cardiovascular related deaths (16), but the coronary uptake of [^64^Cu]Cu-DOTATATE has never been assessed before. Therefore, in the present pre-specified sub-study of the LIRAFLAME trial we aimed at investigating the potential effects of liraglutide on coronary vessel inflammation measured as the uptake of [^64^Cu]Cu-DOTATATE in participants with type 2 diabetes.

## Materials and Methods

### Design and Participants

This is a sub-study of the LIRAFLAME trial, a double-blinded randomized controlled clinical trial of 102 individuals with type 2 diabetes (14).

This pre-specified sub-study consisted of 30 individuals with type 2 diabetes who had previously been included, and undergone randomization procedures, as part of the main trial. Uptake of [^64^Cu]Cu-DOTATATE in the carotid arteries from the same patients has previously been published (15). Participants were randomly included from the main trial based on availability of the radiotracer [^64^Cu]Cu-DOTATATE and willingness to participate in the present study. All participants fulfilled the major inclusion criteria of age ≥ 50 years, HbA_1c_ ≥ 48 mmol/mol and eGFR ≥ 30 mL/min/1,73 m^2^. Treatment with cholesterol- and glucose lowering medications had to be stable for a minimum of 4 weeks prior to enrollment. Notable major exclusion criteria were type 1 diabetes, cancer or any other clinically relevant disorder not associated with type 2 diabetes history. Treatments 90 days prior to enrollment with glucocorticoids, calcineurin inhibitors, GLP1-RA’s and other agents which in the investigators’ opinion might interfere with the effect of the study-drug. Full list of in- and exclusion criteria has previously been published (14).

All participants were, as part of the main trial, randomized to either treatment with liraglutide (n=15) or placebo (n=15) for a period of 26 weeks. Dosing regimen was set up in a dose escalating manner with 0.6 mg once daily for 1 week, followed by 1.2 mg once daily for 1 week, followed by 1.8 mg once daily for the remainder of the trial. The dosing regimen was flexible throughout the study, and all participants were kept on the highest tolerated dose.

At baseline, information on cardiovascular risk factors was collected and analysis of serum lipids, plasma creatinine, systolic blood pressure, high-sensitivity C-reactive protein (hs-CRP) and HbA_1C_ was performed using standard methods.

The study was approved by the local ethics committee (H-16044546) and the Danish Medicines Agency (2016110109) and was in compliance with the principles of the Declaration of Helsinki. Trial registration: EU Clinical Trials Register (2016-001523-31) and ClinicalTrials.gov (NCT03449654).

### Imaging

Participants underwent both [^64^Cu]DOTATATE PET/CT and CT for calculation of CACS at both baseline and at end of treatment using an integrated Siemens Biograph mCT64 system (Siemens, Berlin, Germany).

The CT CACS scan (120 kVp, mAs 70) was performed during an end inspiratory breath hold with electrocardiography (ECG)-gating, axial mode, collimation 8 x 3.0 mm.

Static [^64^Cu]Cu-DOTATATE PET images covering the heart were acquired 60 minutes from time of injection of 200 MBq of [^64^Cu]DOTATATE in three-dimensional list mode. A low dose CT (120 KeV, mAs 40) with a slice-thickness of 3 mm was performed for the purposes of attenuation correction and anatomical localization of the heart and epicardial coronary arteries.

A routinely used, optimized clinical reconstruction protocol of the PET scan using CT based attenuation correction was employed, with both resolution-recovery (point spread function, TrueX) and time-of-flight (2 iterations, 21 subsets, zoom 1.0) giving 400x400 image slices (voxel size 2.00x2.04x2.04). A 2 mm full width at half maximum Gaussian filter was applied to all images post-reconstruction.

### Image Analysis

Co-registered axial PET/CT images were analyzed by observers blinded to all clinical data (including treatment group) and time-point using the Osirix MD imaging platform (version 11.0.01, Pixmeo, Bernex, Switzerland). Two dimensional (2D) Regions of interest (ROIs) were drawn around the artery wall boundary on all major epicardial coronary arteries on axial slices of the fused PET/CT scans. A total of 715 ROIs were drawn on 236 individual coronary artery segments. From the 2D ROI’s, both maximum standardized uptake values (SUV_max_) were measured and a mean-of-the-maximum value was calculated (mSUV_max_). SUV_max_ and mSUV_max_ values are reported both on a participant level and for each individual coronary segment.

CACS quantification was performed *ad modum* Agatston using dedicated software (Syngo.via, Siemens Healthcare, Erlangen, Germany) (17). A total CACS value was calculated by summation of each coronary artery segment (left main, left anterior descending, circumflex artery and the right coronary artery). CACS was not calculated on participants who had previously undergone coronary artery bypass surgery, pacemaker implantation or coronary stenting. CACS was analyzed in a total of 25 participants (n=11 from the liraglutide group and n=14 from the placebo group). CACS data from the all participants in the LIRAFLAME trial has been published previously (14). [^64^Cu]Cu-DOTATATE SUV uptake values from the carotid arteries of the participants in this sub-study has previously been published as part of another trial (15).

PET/CT imaging data collected at end-of-treatment from one participant in the placebo group was unreadable due to a technical issue. Data from this participant were therefore not included in the final analysis.

### Statistical Analysis

Statistical analysis was performed using the programming language R version 4.02 (R Foundation, Vienna, Austria) with the integrated development environment RStudio version 1.3.1056 (RStudio, Boston, USA). The outcome of interest in this study was comparison of the uptake value of [^64^Cu]Cu-DOTATATE in the coronary arteries between the intervention group receiving treatment with liraglutide and the placebo group, and is reported as change in uptake values from baseline to end of treatment.

Normally distributed continuous variables are expressed as means with standard deviation (SD) or 95% confidence interval (95%CI), and compared with Student’s t-test where appropriate. Non-normally distributed variables are presented as medians with inter-quartile range (IQR) and compared using Wilcoxon’s signed rank test. Categorical variables are expressed as numbers and percentages and analyzed with χ^2^-test or Fisher’s exact test, where appropriate. Uptake values from the analysis of individual coronary segments were analyzed with the use of a mixed-effect model with a repeated measures approach, where allocation to treatment or placebo (group) was used as a fixed effect and participant ID was treated as a random effect. A value of 0.1 was added to the CACS values, which were then log_2_-transformed before further analysis.

Correlation analysis on normally distributed baseline data was performed using linear regression and Pearson’s correlation coefficient. Correlations for non-normally distributed data were assessed using the Kendall rank correlation coefficient.

No formal sample size calculation was performed prior for this sub-study, although a power calculation was conducted prior to the main trial to establish the needed sample size for primary end point analysis (14).

Two-sided p-values < 0.05 were considered significant.

## Results

### Participants

Baseline characteristics were generally similar in the two treatment groups. Only triglycerides differed significantly between the two groups (p=0.01). The sub-study population was predominantly males older than 60 years with a body mass index above 25. Ninety percent of all the patients were prescribed a lipid lowering agent, and total cholesterol, LDL cholesterol, HDL cholesterol, and CACS were similar in the two groups (**Table 1**).

**Table 1:** Baseline clinical characteristics.

	Total (n = 30)	Liraglutide (n = 15)	Placebo (n = 15)	p-value
Age – years	66.4 (7.2)	65.9 (8.3)	66.9 (6.3)	0.69
Sex – Female (%)	5 (16.7%)	2 (13.3%)	3 (20%)	1.0
Body mass index (kg/m^2^)	28.9 (4.3)	29.5 (4.0)	28.2 (4.7)	0.41
Cardiovascular risk factors				
Total cholesterol (mmol/L)	4.2 (0.75)	4.4 (0.7)	4.0 (0.8)	0.25
LDL cholesterol (mmol/L)	2.2 (0.51)	2.2 (0.54)	2.1 (0.49)	0.70
HDL cholesterol (mmol/L)	1.3 (0.44)	1.2 (0.47)	1.3 (0.43)	0.50
Triglycerides (mmol/L)	1.7 (0.88)	2.1 (0.97)	1.3 (0.55)	0.01
Systolic blood pressure (mmHg)	134 (19)	133 (14)	136 (24)	0.68
Hypertension (%)	22 (73.3%)	12 (80.0%)	10 (66.7%)	0.68
Smoking - current or ex (%)	28 (93.3%)	14 (93.3%)	14 (93.3%)	1.0
10-year Framingham risk score (%)	30.3 [24.8 – 38.2]	30.3 [24.6 – 39.1]	30.4 [25.2 – 36.2]	0.93
Type 2 diabetes				
HbA_1c_ (mmol/mol)	56.4 (9.2)	59.1 (10.4)	53.7 (7.0)	0.11
Duration (years)	12.3 [5.7 – 19.8]	13.9 [5.9 – 20.9]	8.8 [5.5 – 17.2]	0.43
Insulin use – current (%)	12 (40.0%)	8 (53.3%)	4 (26.7%)	0.14
SGLT2 inhibitor treatment (%)	6 (20.0%)	3 (20.0%)	3 (20.0%)	1.0
Previous cardiovascular disease*	8 (26.7%)	6 (40.0%)	2 (13.3%)	0.21
Cardiovascular medications				
Lipid lowering medication (%)	27 (90.0%)	14 (93.3%)	13 (86.7%)	1.0
Aspirin (%)	10 (33.3%)	4 (26.7%)	6 (40.0%)	0.44
Estimated glomerular filtration rate (mL/min/1.73m^2^)	86.5 [81.0 – 90.0]	86.0 [81.0 – 89.5]	87.0 [78.5 – 90.0]	0.92
Coronary artery calcium score (Agatston units)	65 [9 - 239]	30 [12 - 208]	108 [8 – 232]	0.83
High sensitivity C-reactive protein (mg/L)	1.4 [0.9 – 3.1]	1.4 [1.0 – 3.2]	2.0 [0.8 – 2.8]	0.88

Data are presented as mean (SD), n (%) or median [IQR]. Most of the baseline values have previously been published (15).

*Previous cardiovascular disease was defined as previous stroke, myocardial infarction, percutaneous coronary intervention, coronary artery bypass graft, claudication, peripheral arterial thrombosis and/or angina pectoris requiring nitroglycerin treatment.

HbA_1c_, hemoglobin A1c; LDL, low-density lipoprotein; HDL, High-density lipoprotein; SGLT2, sodium glucose transporter 2.

A significant reduction in the mean weight, from baseline until end-of-treatment, in the group treated with liraglutide was observed compared to the placebo group (mean change in body-weight [95%CI]: Liraglutide: -3.0 [-4.7 to 1.2] kg vs. placebo: -0.3 [-1.1 to 0.6] kg; p = 0.008). HbA_1c _from baseline until end-of-treatment was also significantly reduced in the liraglutide treated group compared to the placebo group (mean change in HbA1c: Liraglutide: -6.1 [-10.1 to -2.1] mmol/mol vs. placebo: 0.1 [-2.6 to 2.9]; p = 0.005). No significant changes were observed between the two groups with regards to markers of cardiovascular disease such as serum-lipid profile (total-cholesterol, LDL cholesterol, HDL cholesterol and triglycerides), hs-CRP, systolic blood pressure or CACS (**Table 2**).

**Table 2:** Change from baseline to follow-up in markers of cardiovascular disease and inflammation.

	Mean (95%CI) or median [IQR] change from baseline	p-value
Total cholesterol (mmol/L)		
Liraglutide (n = 15)	-0.35 (-0.68, -0.01)	0.30
Placebo (n = 14)	-0.13 (-0.42, 0.16)	
LDL cholesterol (mmol/L)		
Liraglutide (n = 14)	-0.26 (-0.48,-0.05)	0.85
Placebo (n = 14)	-0.29 (-0.53, -0.058)	
HDL cholesterol (mmol/L)		
Liraglutide (n = 15)	0.003 (-0.099, 0.105)	0.12
Placebo (n = 14)	0.151 (-0.021, 0.324)	
Triglycerides (mmol/L)		
Liraglutide (n = 15)	-0.07 (-0.72, 0.58)	0.76
Placebo (n = 14)	0.04 (-0.29, 0.36)	
Systolic blood pressure		
Liraglutide (n = 15)	6.7 (1.8, 11.6)	0.39
Placebo (n = 14)	3.0 (-4.9, 10.9)	
Coronary artery calcium score (Agatston units)		
Liraglutide (n = 11)	1 [0 to 10]	0.62*
Placebo (n = 13)	2 [0 to 31]	
High sensitivity C-reactive protein (mg/L)		
Liraglutide (n = 15)	-0.20 [-0.46 to 0.41]	0.46*
Placebo (n = 14)	-0.08 [-0.45 to 0.14]	
HbA_1c_ (mmol/mol)		
Liraglutide (n = 15)	-6.1 (-10.1, -2.1)	0.005
Placebo (n = 14)	0.1 (-2.6, 2.9)	
Body weight (kg)		
Liraglutide (n = 15)	-3.0 (-4.7, 1.2)	0.008
Placebo (n = 14)	-0.3 (-1.1, 0.6)	

Data are presented as mean (95% confidence interval) or median [IQR]. P-values are calculated using unpaired t-test or Wilcoxon signed-rank test for comparison of change from baseline until end of treatment between the 2 groups.

CACS, coronary artery calcium score; HDL, high-density lipoprotein; Hs-CRP, high-sensitivity c-reactive protein; LDL, low-density lipoprotein.

*Hypothesis test calculated using log_2_-transformed value.

Change values for body-weight, LDL cholesterol, Hs-CRP, systolic blood pressure and HbA_1C_ in the liraglutide group has been published previously (15).

### Effect of Liraglutide on [^64^Cu]Cu-DOTATATE Uptake in Coronary Arteries

**Figure 1:**
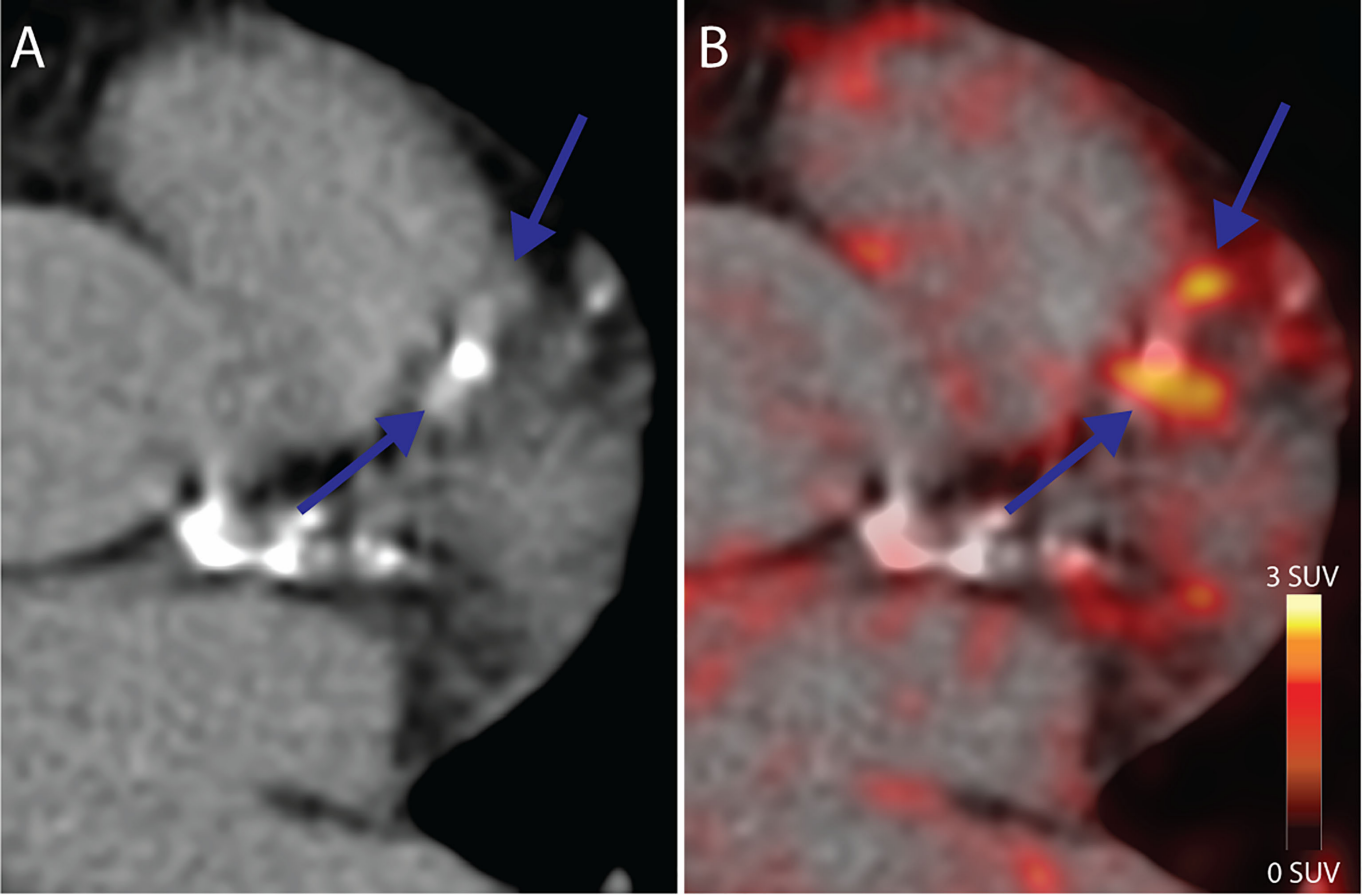
Coronary PET/CT [^64^Cu]Cu-DOTATATE imaging. **(A)** CT Image from a 64-year old male at baseline showing calcified lesions of the left anterior descendant artery with focal **(B)** [^64^Cu]Cu-DOTATATE uptake on PET (blue arrows) bordering the calcified lesions. CT, computed tomography; PET, positron emission tomography.

Uptake of [^64^Cu]Cu-DOTATATE in the myocardium was low enough in all participants to allow for PET uptake measurements in the coronary arteries, as exemplified in **Figure 1**.

**Figure 2:**
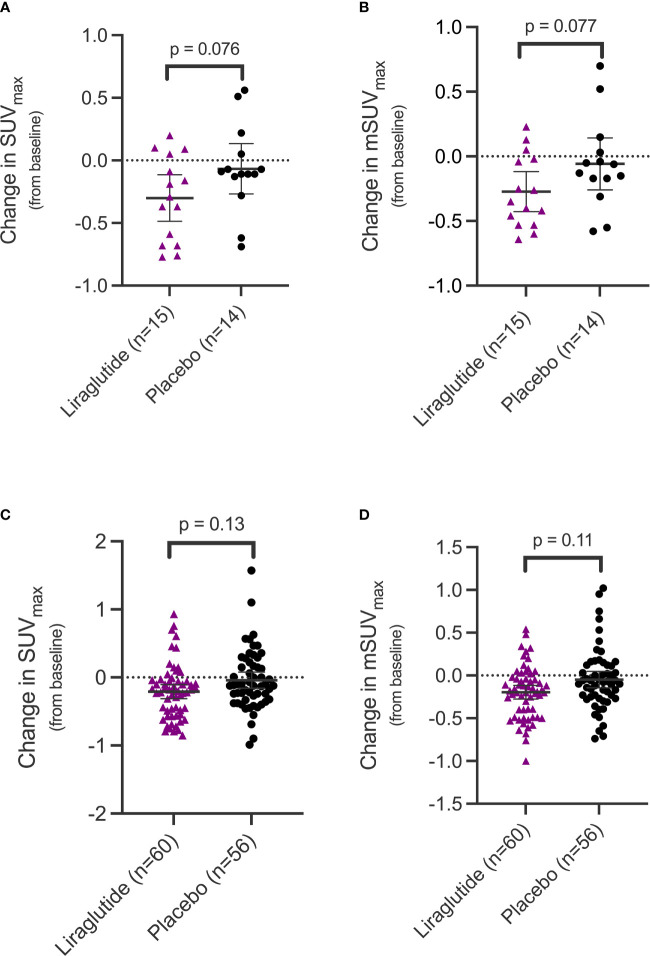
Change in uptake values of [^64^Cu]Cu-DOTATATE in the coronary arteries in the two treatment groups. SUV_max_ **(A)** and mSUV_max_ **(B)** values in the coronary arteries at the participant level and SUV_max_ **(C)** and mSUV_max_ **(D)** on the coronary segment level. Error bars indicate 95% confidence interval. SUV_max_, maximum standardized uptake value; mSUV_max_, mean of the maximum standardized uptake value.

The outcome of interest, coronary artery inflammation, assessed as change in SUV_max_ and mSUV_max_ from baseline until end-of-treatment showed a significant decrease in the liraglutide treated group at the participant level (SUV_max_ [95%CI]: -0.30 [-0.53 to -0.07] g/ml, p=0.013; mSUV_max_: -0.27 [-0.45 to -0.095] g/ml, p=0.004), but not in the placebo treated group (SUV_max_: -0.067 [-0.41 to 0.28] g/ml, p=0.69; mSUV_max_: -0.058 [-0.33 to 0.22] g/ml, p=0.67). A borderline significant trend was observed when comparing the mean change in SUV_max_ and mSUV_max_ between the 2 groups (SUV_max_: -0.23 [-0.49 to 0.03] g/ml, p=0.076; mSUV_max_: -0.21 [-0.46 to 0.03] g/ml, p=0.077) (**Figures 2A, B**).

At the coronary segment level, we also observed a significant change in the liraglutide group from baseline until end-of-treatment (SUV_max_: -0.21 [-0.31 to -0.11] g/ml, p=0.001; mSUV_max_: -0.20 [-0.28 to -0.12] g/ml, p < 0.0001) but not in the placebo group (SUV_max_: -0.04 [-0.15 to 0.07] g/ml, p=0.49; mSUV_max_: -0.05 [-0.13 to 0.04], p=0.30). As with the results at the participant level, a non-significant trend towards a difference was observed when comparing the mean difference between change in uptake between the two groups at the coronary segment level (SUVmax: - 0.17 [-0.39 to 0.05] g/ml, p=0.13; mSUVmax: -0.15 [-0.34 to 0.04] g/ml, p=0.11) (**Figures 2C, D**).

**Figure 3:**
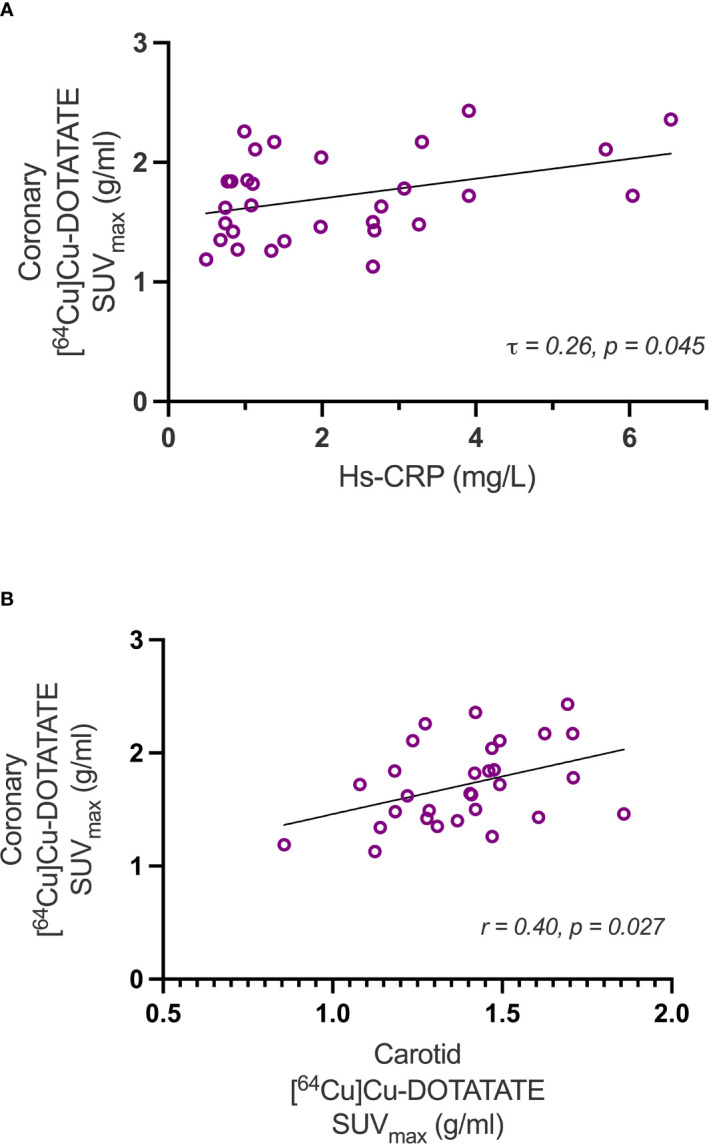
Correlations with Hs-CRP and carotid artery [^64^Cu]Cu-DOTATATE uptake. The scatter-plots show correlations of coronary inflammation measured as [^64^Cu]Cu-DOTATATE SUV_max_ uptake versus hs-CRP **(A)** and carotid SUV_max_ **(B)**. Hs-CRP, high sensitivity c-reactive protein.

Uptake values at the participant level (SUV_max_) measured at baseline showed a weak positive correlation with the marker of systemic inflammation, hs-CRP (τ = 0.26; p = 0.045) (**Figure 3A**). Also, coronary and carotid artery [^64^Cu]Cu-DOTATATE SUV_max_ of the same participants was positively correlated (r=0.40, p= 0.027) (**Figure 3B**).

## Discussion

Mechanisms of cardiovascular risk reduction observed with GLP-1 RA treatment has previously been proposed to be, in part, mediated by an effect on the atherosclerotic process (7, 10).

In this sub-study of 30 participants with type 2 diabetes, from the randomized double blinded placebo controlled LIRAFLAME trial, we observed a small but significant reduction in [^64^Cu]Cu-DOTATATE uptake in the coronary arteries in the liraglutide treated group and not in the placebo group after 26-weeks of treatment, although the difference in change in uptake between the two groups did only reach borderline statistical significance (p=0.08).

The need for accurate non-invasive risk stratification to guide interventions among populations with increased risk of cardiovascular disease is currently growing. Multimodality PET imaging has proven to be a powerful approach in the evaluation of atherosclerotic disease and the effect of anti-atherosclerotic therapies (4, 18). Especially the two radiotracers [^18^F]FDG and [^18^F]NaF, imaging vessel metabolic activity and micro-calcifications, respectively, have been heavily investigated in this context over the recent years. In the main LIRAFLAME trial, we observed no effect of liraglutide on vascular inflammation in the carotid arteries and aorta assessed using [^18^F]FDG PET in 102 participants with type 2 diabetes (14).

In this sub-study, we utilized the novel radiotracer [^64^Cu]Cu-DOTATATE targeting the SSTR_2_ receptor found on the surface of activated M1 macrophages, in the coronary arteries (19, 20). This enables direct *in vivo* imaging and quantification of the inflammatory burden in atherosclerotic disease. So far, SSTR_2_ imaging with relation to atherosclerotic disease has primarily been investigated in retrospective studies (21–24), but one cross sectional study has evaluated its use in coronary arteries, and found that [^68^Ga]Ga-DOTATATE correctly identified culprit arteries and stable plaques with high risk CT features (5). The investigators also found that [^68^Ga]Ga-DOTATATE performed superiorly to [^18^F]FDG when imaging coronary arteries, both due to the specificity of the tracer in identifying macrophages compared to nonspecific metabolic activity and less myocardial spill-over, offering improved interpretability of the scans. Our results indicate that coronary imaging with [^64^Cu]Cu-DOTATATE is also feasible and correlates to both a systemic marker of inflammation and carotid artery uptake of [^64^Cu]Cu-DOTATATE. The advantage of the radiotracer used by us, [^64^Cu]Cu-DOTATATE, compared to [^68^Ga]Ga-DOTATATE, is the better spatial resolution due to a four-fold lower positron range of ^64^Cu (1 mm) compared to ^68^Ga (4 mm). As coronary arteries are relatively small compared to the positron range, it may be of particular importance for imaging these vessels.

The findings from the analysis of the coronary arteries in the present study are in accordance with the previously published results from another sub-study performed in the LIRAFLAME cohort assessing carotid artery inflammation also using [^64^Cu]Cu-DOTATATE (15) where a significant effect, when comparing the uptake values in the liraglutide group from baseline with end-of-treatment was observed, but no significant difference was found between the liraglutide treated group and the placebo group. This is also in accordance with the observed correlation between baseline coronary SUV_max_ values and carotid SUV_max_ values from the same participants. Both studies suggest that [^64^Cu]Cu-DOTATATE PET offers a more specific and sensitive method for evaluation of vascular inflammation due to macrophage activity, compared to that of [^18^F]FDG PET. [^64^Cu]Cu-DOTATATE PET was able to detect a difference in uptake in the treated group, even though the included participants were not selected based on an increased cardiovascular risk beyond that of type 2 diabetes itself, as was the case in many of the larger trials investigating the effects of GLP1-RA’s on cardiovascular outcomes (7, 9, 10, 25). Also, [^64^Cu]Cu-DOTATATE has the advantage, compared to [^18^F]FDG, of being independent of blood glucose values, which might be a factor to consider when imaging a diabetic population (26).

Baseline values of coronary [^64^Cu]Cu-DOTATATE uptake also significantly correlated with hs-CRP values, a marker of low-grade systemic inflammation, which also testifies to the systemic involvement in atherosclerotic disease (27, 28).

GLP-1 RA’s have a wide range of effects in different organ systems, and both preclinical and clinical trials have sought to uncover the mechanisms underlying the observed cardioprotective effect of this class of drugs. The primary effects of the GLP-1RA’s are reduction in blood-glucose and body weight, which were also observed in the liraglutide treated group of this study. Preclinical evidence suggests that the proposed anti-atherosclerotic effects of these drugs might be attributable to mechanisms other than body weight and glucose lowering alone. In an animal study utilizing the long acting GLP-1RA semaglutide at doses that did not lower body weight, an anti-atherosclerotic effect was still observed (11). The evidence regarding the effect of GLP-1RA’s on macrophages in atherosclerosis is somewhat disputed. Some preclinical data suggests that GLP-1RA’s have no effect of the deposition of macrophages in the vessel-wall during atherosclerotic conditions (13, 29). Whereas other studies suggest that a reduction in macrophage content plays a central role in the atheroprotective mechanisms of GLP-1RA’s (12, 30, 31), along with a modulation of macrophage phenotype and pro-inflammatory signaling (32, 33).

### Limitations

The main limitation of this study was that it was conducted in a sub-population of the original trial cohort and therefore not powered to detect differences between the two groups.

A low-dose ECG gated attenuation CT-scan was used for anatomical localization of the coronary arteries. A contrast enhanced coronary CT-angiography may have provided improved localization of the coronary arteries.

## Conclusion

This study demonstrated a significant reduction in [^64^Cu]Cu-DOTATATE uptake in the coronary arteries in the liraglutide treated group and not in the placebo group after 26-weeks of treatment. This study demonstrates that [^64^Cu]Cu-DOTATATE PET/CT as a marker of coronary inflammation is feasible and quantifiable, and uptake correlates with the systemic inflammation marker hs-CRP.

## Data Availability Statement

Anonymized data can be obtained from the corresponding author upon reasonable request. Necessary data protection agency and ethical committee approvals must be provided in compliance with relevant legislation.

## Ethics Statement

The study was approved by the local ethics committee (H-16044546) and the Danish Medicines Agency (2016110109) and was in compliance with the principles of the Declaration of Helsinki. The patients/participants provided their written informed consent to participate in this study.

## Author Contributions

JJ, EZ, RR, BS, TH, AK, and PR contributed to the study design and data interpretation. JJ, EZ, RR, VC, and TH acquired data. EZ recruited participants. JJ performed statistical analysis. JJ drafted the manuscript. All authors contributed to the article and approved the submitted version.

## Funding

The study was funded by Novo Nordisk A/S and Skibsreder Per Henriksen, R. og hustrus fund. Steno Diabetes Center Copenhagen, Rigshospitalet, and Cluster for Molecular Imaging, University of Copenhagen, Denmark have provided internal funding.

## Conflict of Interest

AK has received consultancy fees from Novo Nordisk and is an inventor on a patent of [64Cu]Cu-DOTATATE. RR, BS, TH, and PR have shares in Novo Nordisk A/S. BS and EZ are now employees of Novo Nordisk A/S. PR has received the following: Consultancy and/or speaking fees (to Steno Diabetes Center Copenhagen) from AbbVie, Astellas, AstraZeneca, Bayer, Boehringer Ingelheim, Bristol-Myers Squibb, Eli Lilly, MSD, Novo Nordisk and Sanofi Aventis; Research grants to institution from AbbVie, AstraZeneca and Novo Nordisk.

The remaining authors declare that the research was conducted in the absence of any commercial or financial relationships that could be construed as a potential conflict of interest.

## Publisher’s Note

All claims expressed in this article are solely those of the authors and do not necessarily represent those of their affiliated organizations, or those of the publisher, the editors and the reviewers. Any product that may be evaluated in this article, or claim that may be made by its manufacturer, is not guaranteed or endorsed by the publisher.
